# The fabrication and growth mechanism of AlCrFeCoNiCu_0.5_ HEA thin films by substrate-biased cathodic arc deposition

**DOI:** 10.1038/s41598-022-26232-9

**Published:** 2023-01-05

**Authors:** Hong Zhao, Zhong Zheng, Behnam Akhavan, Kostadinos Tsoutas, Lixian Sun, Haoruo Zhou, Marcela M. Bilek, Zongwen Liu

**Affiliations:** 1 School of Chemical and Biomolecular Engineering, The University of Sydney, Sydney, NSW 2006 Australia; 2 The University of Sydney Nano Institute, The University of Sydney, Sydney, NSW 2006 Australia; 3 School of Physics, The University of Sydney, Sydney, NSW 2006 Australia; 4 School of Biomedical Engineering, The University of Sydney, Sydney, NSW 2006 Australia; 5 School of Engineering, University of Newcastle, Callaghan, NSW 2308 Australia; 6 Guangxi Key Laboratory of Information Materials, Guangxi Collaborative Innovation Center of Structure and Property for New Energy and Materials, School of Material Science & Engineering, Guilin University of Electronic Technology, Guilin, 541004 People’s Republic of China; 7 School of Aerospace, Mechanical and Mechatronic Engineering, The University of Sydney, Sydney, NSW 2006 Australia

**Keywords:** Engineering, Materials science, Nanoscience and technology

## Abstract

AlCrFeCoNiCu_0.5_ thin films were fabricated by cathodic arc deposition under different substrate biases. Detailed characterization of the chemistry and structure of the film, from the substrate interface to the film surface, was achieved by combining high-resolution transmission electron microscopy, X-ray photoelectron spectroscopy, and atomic force microscopy. Computer simulations using the transport of ions in matter model were applied to understand the ion surface interactions that revealed the key mechanism of the film growth. The final compositions of the films are significantly different from that of the target used. A trend of elemental segregation, which was more pronounced with higher ion kinetic energy, was observed. The XPS results reveal the formation of ${\mathrm{Al}}_{2}{\mathrm{O}}_{3}$ and ${\mathrm{Cr}}_{2}{\mathrm{O}}_{3}$ on the thin film surface. The grain size is shown to increase with the increasing of the ion kinetic energy. The growth of equiaxed grains contributed to the formation of a flat surface with a relatively low surface roughness as shown by atomic force microscopy.

## Introduction

High-entropy alloys (HEAs) are defined as alloy systems comprising at least 5 elements in equal or nearly equal atomic concentration, ranging from 5at.% to 35at.%^1^. They were first proposed by Yeh et al.^1^ and Cantor et al.^2^ in 2004. HEAs tend to form as a solid solution phase, with crystalline phases that can include face-centered cubic (FCC), body-centered cubic (BCC) and hexagonal close packing (HCP) rather than pure intermetallic phases^3–5^. Due to the influences of the high entropy effect, lattice distortion and sluggish diffusion^1^, HEAs demonstrate desirable mechanical, chemical and physical properties, such as high yield strength^6–8^, high fracture toughness^9,10^, high resistance to oxidation^11,12^ and corrosion^13–15^ and thermal stability^16^, depending on their chemical compositions. While bulk HEA materials are well studied, there is little understanding of the deposition mechanisms of thin film HEA samples, particularly those produced by cathodic arc plasmas.

The growth behavior and properties of HEA thin films strongly depend on the synthesis technique. A range of techniques, such as magnetron sputtering, laser cladding, thermal spraying, electrochemical deposition and vacuum arc deposition, have been applied to deposit HEA thin fims^17^. The fabrication technologies of HEA bulk samples usually undergo slow cooling rates, while HEA thin films and coatings experience rapid cooling during synthesis due to their physical dimensions and production methods^17^. This rapid quenching effect leads to the preferential formation of solid solution phases and nanocrystalline structures^18,19^. Significant research into HEA thin films has been focused on their applications as thermal, mechanical and chemical surface protection coatings^20,21^.

Here we report the fabrication of AlCrFeCoNiCu_0.5_ thin films by cathodic arc deposition. Cathodic arc deposition is a physical vapor deposition (PVD) technology involving the cathode material being vaporized and ionized by the electric arc. The ions then condense on a substrate to form a thin film. The advantages of using cathodic arc deposition are its high deposition rate, high ionization efficiency of the cathode material, and controllable ion kinetic energy ranging around 25–100 eV (without substrate bias). The energy control of the depositing flux is critical in producing dense films with good adhesion and crystallinity^22,23^. A disadvantage of using cathodic arc deposition, however, is the concurrent production of macroparticles of cathode material within the plasma stream^24^. If these particles are incorporated into the growing film, they usually register negative effects on film quality and properties^25^. A magnetic filter can be installed into a cathodic arc system to solve this issue. The magnetic field created by an elbow-shaped coil can guide the plasma stream around a bend, preventing macroparticles from arriving at the substrate. Since the macroparticles display a much larger mass-to-charge ratio than the ions, they have too much inertia to be deflected around the bend of the magnetic filter.

In this work, high-entropy alloy AlCrFeCoNiCu_0.5_ thin films were fabricated by cathodic arc deposition with applied substrate biases of 0 V, −50 V, and −100 V. X-ray photoelectron spectroscopy (XPS), atomic force microscopy (AFM) and transmission electron microscopy (TEM) were employed to determine the film structure and chemical composition from the film-substrate interface to the film surface. The mechanisms underpinning the film structure formation were explored with the aid of computer modelling using the transport of ions in matter (TRIM) software package to assess the sputtering, backscattering and range of the ions in the film and substrate. The mean ion kinetic energy input for TRIM simulations of ion surface interactions with different substrate biases was estimated using the cohesive energy rule proposed by Anders et al.^26^ and Brown’s measurements of ion charge state distributions^27^.

## Methodology

### Sample fabrication

AlCrFeCoNiCu_0.5_ thin film samples were fabricated by filtered cathodic arc deposition with three different substrate biases, 0 V, −50 V, and −100 V (Table 1). A single cylindrical cathode from ACI Alloys Inc. with a diameter of 44 mm, a thickness of 36 mm and a purity of 99.95% manufactured by vacuum arc melting was used as the thin film material source. The cathode composition is shown in Table 2. A hollow cylindrical copper anode with an inner diameter of 50.8 mm, an outer diameter of 54 mm and a length of 41 mm was coaxially mounted around the HEA cathode. The anode protruded 12 mm beyond the cathode surface with its outlet facing to the magnetic filter. The magnetic filter, which was used to guide the plasma stream from the cathode surface to the substrate, was a 90-degree copper tube solenoid with 22.5 turns of 140-mm cross-sectional diameter that is bent into an elbow with a radius curvature of 440 mm.

**Table 1:** Mean ion kinetic energy of vacuum arc with or without substrate bias.

Element	Ion kinetic energy with no substrate bias (eV)	Ion kinetic energy with −50 V of substrate bias (eV)	Ion kinetic energy with −100 V of substrate bias (eV)
Al	81	167.5	254
Cr	81	187.5	294
Fe	81	172	263
Co	81	167.5	254
Ni	81	169	257
Cu	81	184	287

**Table 2:** Atomic percentage of the HEA elements in the cathode and thin films deposited with various substrate bias.

	Al	Cr	Fe	Co	Ni	Cu
Stoichiometric cathode at%	18.2	18.2	18.2	18.2	18.2	9.0
Thin film (0 V) at%	6.7 ± 0.7	24.2 ± 4.1	22.0 ± 3.7	20.4 ± 3.5	18.2 ± 3.3	8.5 ± 1.5
Thin film (−50 V)at%	5.7 ± 0.6	21.7 ± 3.7	22.5 ± 3.8	22.1 ± 3.7	19.7 ± 3.3	8.3 ± 1.4
Thin film (−100 V) at%	6.7 ± 0.5	23.5 ± 4.0	22.1 ± 3.8	20.8 ± 3.6	18.4 ± 3.3	8.4 ± 1.5

Commercial monocrystalline Si wafers with a (100) orientation were used as substrates onto which the films were deposited. The substrates were cut to 80 mm × 24 mm using a tungsten pen and cleaned in a sonicator for 15 min with acetone, ethanol and deionized water being applied sequentially before being loaded onto the substrate holder inside the cathode arc vacuum chamber. The chamber was then pumped to a base pressure of 8.0 × ${10}^{-6}$ mbar.

During deposition, the triggering voltage pulse that initiated the cathodic arc discharge was applied at a frequency of 5 Hz and the discharge pulse length was set to 800 μs. The arc current and duct current were set at 650 A and 350 A, respectively, and were monitored by an oscilloscope, while the DC substrate biases were set to 0 V, −50 V, and −100 V when running the arc pulse. To account for the effect of ion etching which reduces the thin film thickness due to energetic ion bombardment at higher biases, the cathodic arc current pulses were applied at 10,000 for 0 V, 30,000 for −50 V, and 30,000 for −100 V.

### The TRIM modelling

TRIM modelling was carried out to assess the sputtering, backscattering, and the range of the ions in the film and substrate. Our model assumed an ion charge distribution as defined by Brown^27^. The HEA film was simulated as an ideal AlCrFeCoNiCu_0.5_ layer with a stoichiometry of 2:2:2:2:2:1. Each element being implanted was simulated separately, with the ion energy being taken as the weighted energy based on the relevant ion charge state distribution (Table 1). Each ion implantation event was simulated 10,000 times to achieve a good statistical distribution of ion interaction cascades. The displacement energy of each species in the HEA film was derived from ASTM E521^28^, and the surface binding energy was calculated^29,30^, while the lattice binding energy was set at 3 eV for all elements. The values used are shown in the appendix. For implantation into SiO_2_, the data was taken directly from the TRIM library, which uses ICRU 49 as its data source. The angle of impact was set at 90 degrees, i.e., normal to the surface. Data recorded from the simulation of the ion implantation into the films included the number of backscattered ions, sputter yield, and mean penetration depth for each ion species implanted.

### Sample characterization

The thicknesses of the HEA thin films were measured using a DektakXT stylus profilometer manufactured by Bruker, USA. The substrates were masked before deposition to provide sharp steps. Twenty measurements were made across the sharp steps on each of the as-deposited samples with a stylus scanning length of 1.5 mm and a load of 5 mN.

The film surface chemistry was investigated by a Thermo K-Alpha X-ray Photoelectron Spectrometer (XPS) system manufactured by the Thermo Fisher Scientific USA with an X-ray scanning spot size of 400 μm. The photoelectron peak associated with each element was scanned 30 times with a pass energy of 50 eV, dwell time of 50 ms, and energy step size of 0.1 eV. We chose 2p orbital peaks for Cr, Fe, Co, Ni, and Cu, and 2 s orbital peaks for Al for scanning because the signal of Al2p orbital overlaps with that of Cu3p.

Atomic Force Microscopy (AFM) was utilized to perform topography measurements for surface roughness analysis of the HEA thin films. Scans of a 1 μm × 1 μm areas were performed by a Bruker AFM microscope in contact mode. The amplitude setpoint voltage and scanning rate were set at 250 mV and 1 Hz, respectively. Data collected from the microscope was processed by NanoScope (Version 1.9) analysis software to generate three-dimensional (3D) topography of the scanned area and to calculate the average surface roughness.

Scanning transmission electron microscopy (STEM) and High-resolution Transmission electron microscopy (HRTEM) were used to analyze the elemental compositions and microstructures of the HEA thin films. Wedge-shaped cross-sectional TEM specimens were prepared by tripod polishing and attached on molybdenum grids. The specimens were then milled by argon ions in a Gatan PIPS II. The operating voltage in ion milling started from 4 keV and was gradually lowered to 300 eV for specimen thinning and cleaning. Bright-field (BF) imaging, Electron diffraction pattern (EDP) acquisition, and energy dispersive spectroscopy (EDS) mapping were performed with an FEI Themis Z microscope with a 300 kV accelerating voltage. Structural phase identification and lattice parameter calculations based on EDP and HRTEM images were carried out using the Digital Micrograph software (Version 3.42, Gatan Inc.).

## Results and discussion

### Ion kinetic energy estimation and TRIM modelling

To predict the effect of substrate bias on HEA film deposition rate, chemistry and microstructure, an estimation on how the mean kinetic energy of the ion stream varies with substrate bias is required. The ion energy can be estimated using the following equation:

1 $${E}_{kin}= {E}_{kin,0}+Qe{V}_{Sheath}$$

where ${E}_{kin,0}$ is the ion kinetic energy in the absence of substrate bias, and $Q$ is the mean ion charge state, while $e$ is the elementary charge with ${V}_{Sheath}$ being the voltage across the sheath. The second term of the equation, QeV, represents the kinetic energy of ions contributed by the substrate bias^24^. Before estimating the ion kinetic energy, it is important to note that the plasma parameters, such as arc burning voltage, mean ion charge state, and the corresponding ion kinetic energy, are prominently cathode-material property dependent, i.e., cohesive energy^31^. The cohesive energy rule proposed by Anders^26^ was applied to determine the final kinetic energy based on our cathode properties. The logic is that the cathode cohesive energy determines the arc burning voltage, which further determines the power dissipation with a given arc current. The power dissipation was used to estimate the ion kinetic energy^26^. To simplify the estimation process, the arc burning voltage of the HEA cathode was directly measured, to be 23.7 V, and then the average ion kinetic energy of the multicomponent plasma without a bias was estimated, to be 81 eV. This estimation cannot differentiate each specific element, since the deposition was carried out with an alloy cathode.

In order to estimate the ion kinetic energy with a bias, it requires the charge state distribution of each ion species to be known. The ion charge state distributions derived from Brown’s work^27^ were used. The mean ion kinetic energies calculated for each element with different substrate bias voltages are shown in Table 1.

**Figure 1:**
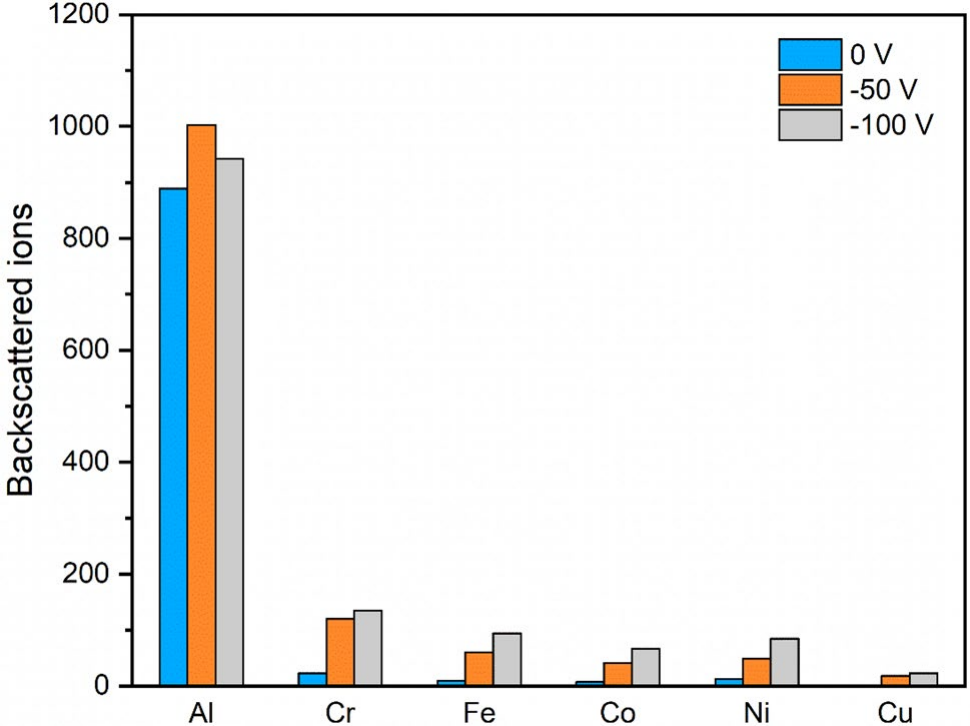
Backscattered ions per 10,000 ions deposited for each element with substrate bias of 0 V, −50 V, and −100 V.

The TRIM results were found to provide a good agreement with our observations of film thickness and deposition rates. Figure 1 shows the number of ions backscattered after interaction with a HEA thin film for simulations for all elements (except Cu) calculated using 10,000 ion impacts and then had its value halved to match the ideal AlCrFeCoNiCu_0.5_ stoichiometry. For aluminum, which possesses a significant mass mismatch with the heavier atoms in the film, it is expected to be backscattered more significantly than the other elements. At −50V, more than 10% of all incoming Al ions would be backscattered, meaning that the deposited films would contain less aluminum when compared to the composition of the ionized particle stream. For the non-aluminum ions, a trend of an increasing amount of backscattering as the bias voltage increased was observed, resulting in lower deposition rates. For aluminum, it was found that the amount of backscattering decreased at the −100 V substrate bias. This is an outcome of both the aluminum having an increased implantation range at this energy (making it harder for deeper ions being back scattered) and a larger fraction of energy being transferred to the recoiled atoms along the implantation trajectory.

**Figure 2:**
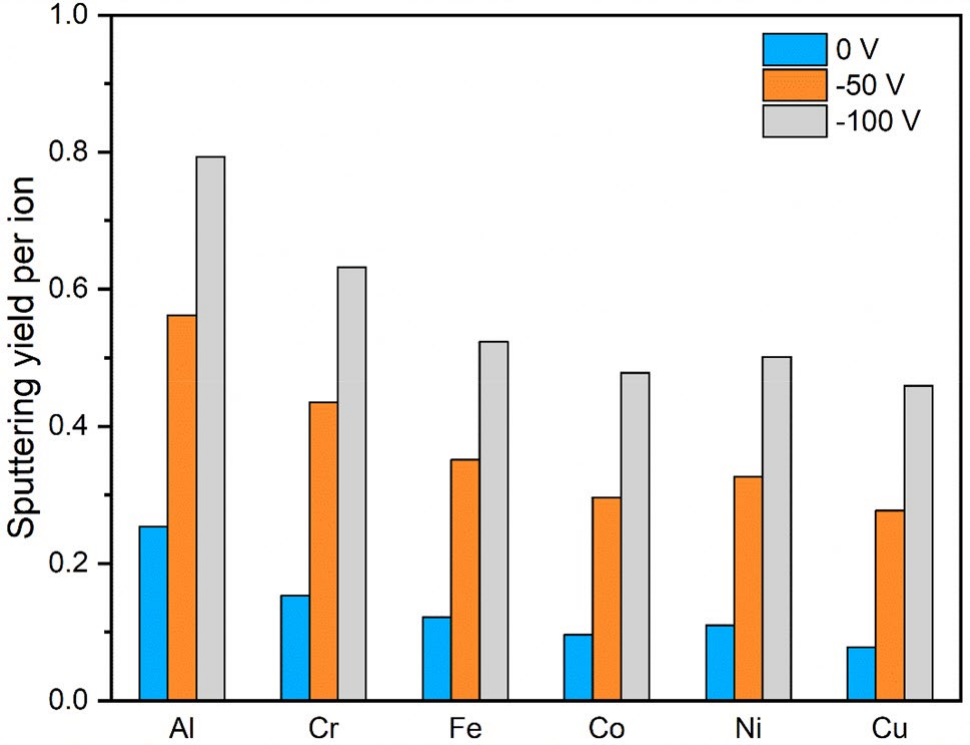
Sputtering yield per ion of deposition for each element with the substrate bias of 0 V, −50 V, and −100 V.

Figure 2 shows the calculated sputter yield which is a measure of how many atoms on average are sputtered from the film for each incoming ion. For each element, the observed trend is that an increase in bias greatly increases the sputter yield, resulting in a decrease in the expected deposition rate.

**Figure 3:**
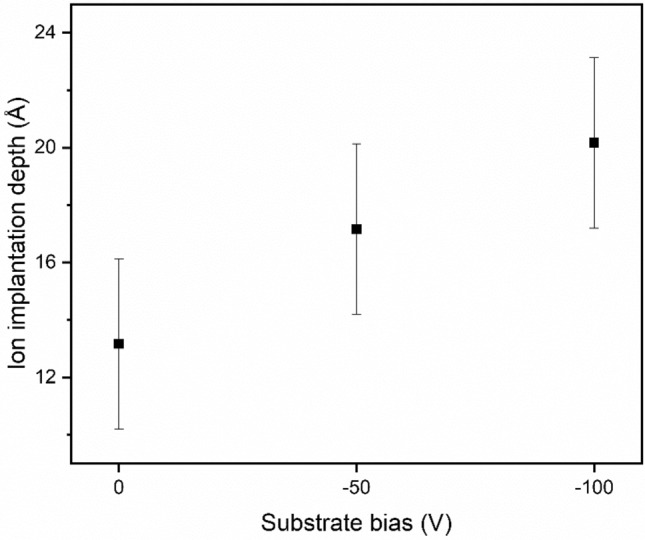
Average ion implantation range as a function of substrate bias for all of the elements into the substrate of ${\mathrm{SiO}}_{2}$.

Ion implantation into the SiO_2_ layer of the Si wafer substrate was simulated to assess the expected ion penetration depths. The data are plotted in Fig. 3. The mean ion implantation range of all elements is presented. It reveals that the ions could penetrate at least 1 nm into the silicon oxide layer, causing an intermixed region of HEA elements at the film’s interface with the SiO_2_. It should be noted that TRIM does not simulate chemical reactions, phase change, or thermal diffusion. Only kinetic interactions between atoms and the implantation target were simulated, that is, the simulation was not dynamic, so the substrate surface always had the constant stoichiometry regardless of how many implantation events had occurred. This suggests that at higher energies, where there is significant atomic motion, rearrangement, and chemical change, the estimated values could be less accurate.

### Thickness and deposition rates

**Figure 4:**
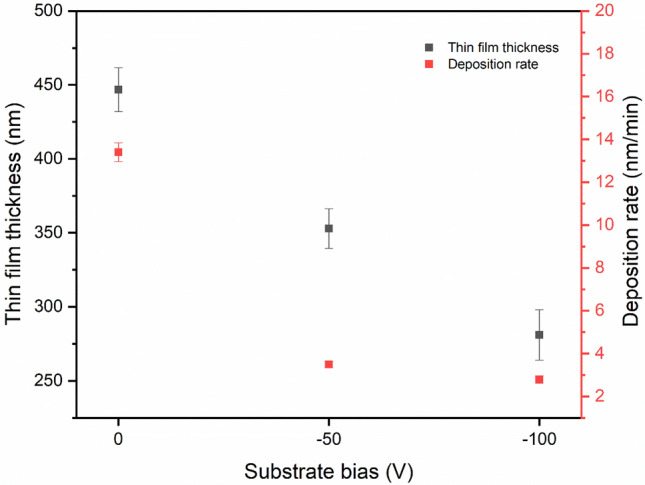
AlCrFeCoNiCu0.5 HEA thin film thickness and deposition rates at various substrate bias.

The relationship between the AlCrFeCoNiCu_0.5_ thin film thickness and the deposition rate with increasing negative substrate bias is shown in Fig. 4. The thickness of film deposited without a substrate bias is 446.8 ± 14.7 nm corresponding to a deposition rate of 13.4 nm/min, whereas the thicknesses of films deposited with substrate biases of −50 V and −100 V are 352.9 ± 13.4 nm and 282.0 ± 17.1 nm corresponding to a deposition rate of 3.5 nm/min and 2.8 nm/min, respectively. A trend of decreasing deposition rate with increasing the negative substrate bias and a significant deposition rate drop between samples fabricated with 0 V and −50 V were observed. This phenomenon is the result of backscattering and self-sputtering during the deposition process. Backscattering during the deposition causes the energetic particles to be reflected from the surface after hitting the substrate, while self-sputtering during the deposition occurs when incoming ions or atoms collide with surface atoms and eject some of them from the film surface^32^.

To explore the nature of the lower deposition rate caused by ion bombardment, it is necessary to clarify the contribution of the different mechanisms during cathodic arc deposition. The dominance of backscattering and self-sputtering, in fact, strongly depends on the incident angle. At normal incidence of the plasma stream to the substrate, the backscattering of ions accounts for a smaller fraction in the order of ${10}^{-2}$, while self-sputtering by ion bombardment contributes more^33^. The TRIM simulation results presented in Figs. 1 and 2 show good agreement with these observations.

### Chemical compositions of the HEA thin films

The elemental composition of each HEA film and its interface with the Si substrate were investigated by STEM-EDS, while the surface oxidation state for each element was evaluated using XPS.

#### Film composition

The elemental composition of the cathode and the as-deposited thin films fabricated using various substrate bias voltages are shown in Table 2. The percentages of constituent atoms in each of the thin films are similar. However, the Al concentration in the thin films is much lower than that of the alloy cathode.

**Figure 5:**
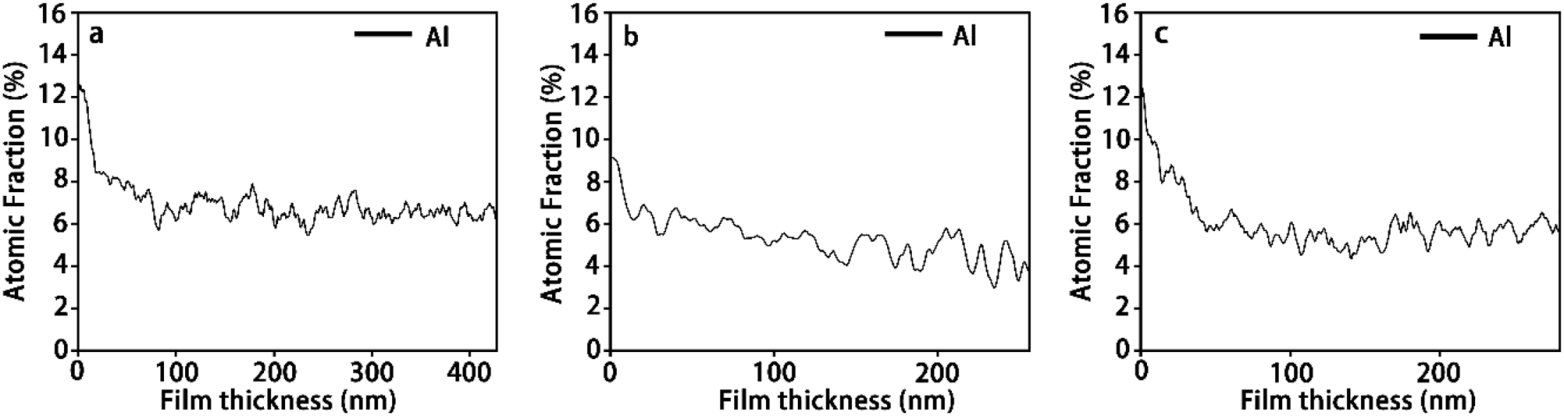
Aluminum atomic fractions along the film thickness from the Si-HEA interface to the thin film surface for (**a**) the thin film with 0 V substrate bias; (**b**) the thin film with −50 V substrate bias; and (**c**) the thin film with −100 V substrate bias. The left side represents the substrate, and the right side represents the thin film surface in each figure.

To understand this phenomenon, the Al concentration profiles are plotted along the thickness from the Si-HEA interface to the HEA thin film surface, as shown in Fig. 5. An enrichment region of Al appeared at the interface, which will be further discussed in the subsequent section on Interface chemistry. Above the interface, the Al concentration declined to an equilibrium level with an atomic fraction of about 6%.

The elemental composition difference between the thin film and the cathode is not dependent on ion kinetic energy but rather on properties of the various atoms since there is no obvious composition change occurring with changing ion energy. The effect of preferential sputtering induced by ion bombardment on the final composition is clear. During the deposition, the as-deposited atoms can be sputtered by incoming energetic ions. The sputtering rate of each element is determined by the atomic mass and the surface binding energy. Those elements, having relatively lower atomic mass and surface binding energy in an alloy system, are energetically preferred to be sputtered. The atomic mass and the estimated value of the surface binding energy of each element based on the crystalline phase, cohesive energy, and elemental concentration are shown in Table 3^29,30^. The crystalline phase determines the number of the nearest neighboring atoms to the target atom. The cohesive energy represents the interaction potential between the atoms. The elemental concentration represents the probability of different pairs that can form with the target atoms. The relatively lower values of the atomic mass and surface binding energy of Al as compared to the other elements in the HEA system are consistent with the TRIM simulation result of higher Al backscattering and sputtering, leading to the lower Al content in each thin film sample relative to that in the target.

**Table 3:** Atomic mass and surface binding energy of each element in an AlCrFeCoNiCu0.5 HEA thin film.

Element	Al	Cr	Fe	Co	Ni	Cu
Atomic mass amu	26.98	52.00	55.85	58.93	58.70	63.55
Surface binding energy eV/atom	4.85	5.33	5.45	5.52	5.55	4.93

#### Interface chemistry

**Figure 6:**
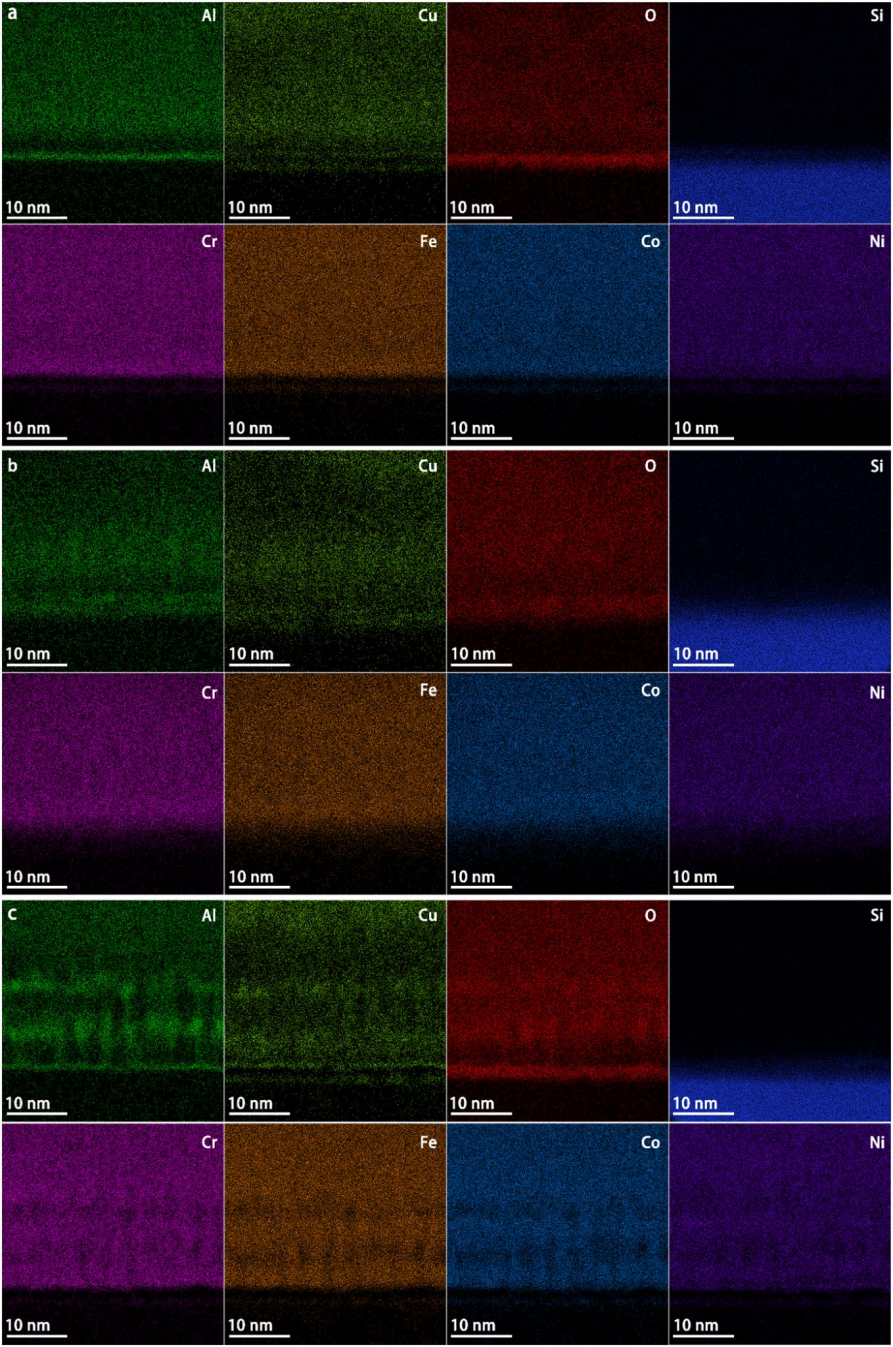
Cross-sectional EDS map of each element at the interface of the AlCrFeCoNiCu0.5 HEA thin films with the substrate at different substrate bias. (**a**) maps with 0 V bias, (**b**) maps with −50 V bias, and (**c**) maps with −100 V bias.

The STEM-EDS maps showing the interface chemistry of the HEA thin films are presented in Fig. 6. The interface between the HEA and SiO_2_ surface oxide layer can be clearly observed in each map. The interaction of deposited atoms with the substrate surface oxygen during the deposition can significantly modify the interfacial chemistry and interfacial bonding. A composition exchange between the HEA elements and Si at the interface was observed. A trend of elemental segregation where Al and Cu were increasingly segregated from the other four elements with increasing substrate bias was also observed above the interface.

In each set of the maps, a layer enriched in aluminum is aligned with a layer enriched in oxygen at the interface, suggesting a region of alumina compound formation. The presence of ${\mathrm{Al}}_{2}{\mathrm{O}}_{3}$ at the interface is consistent with its favorable enthalpy formation of −1676.7 kJ/mol, which is lower than that of all of the other potential oxides. The formation of an ${\mathrm{Al}}_{2}{\mathrm{O}}_{3}$ compound would lead to lowering of the sputter yield of Al near the interface because the surface binding energy of Al in ${\mathrm{Al}}_{2}{\mathrm{O}}_{3}$ is much higher than that in the crystalline HEA^34^. This is likely to explain the higher Al concentration near the interface.

The second important interfacial phenomenon observed is the exchange of HEA elements and Si across the interface. Two mechanisms contributed to this mixing, the Kirkendall diffusion and the energetic ion implantation. Kirkendall diffusion requires mutual solid solubility between the elements involved across the interface region^35^. The deposited atoms cannot choose their impact sites, but they would initially diffuse to form a coherent boundary with the Si substrate towards a configuration that minimized the total Gibbs free energy. During this process, a compressive stress field was accumulated by the continuous ion bombardment. An opposite diffusion of Si and HEA elements was then promoted to relieve the local stress. This diffusion behavior can result in a noncoherent boundary, forming a diffusion layer with a gradual composition change across the interface. Such kind of diffusion layer at the interface usually contributes to good adhesion^35^. Ion implantation can also contribute to the formation of such a mixed layer. With the energies applied, the TRIM modelling suggests that the ions can penetrate 1–2 nm into the SiO_2_ layer, as shown in Fig. 3. The model does not take into account of the thermal motions or diffusion, but only collisional kinetics, so the actual extent of the mixing layer might be underestimated.

**Figure 7:**
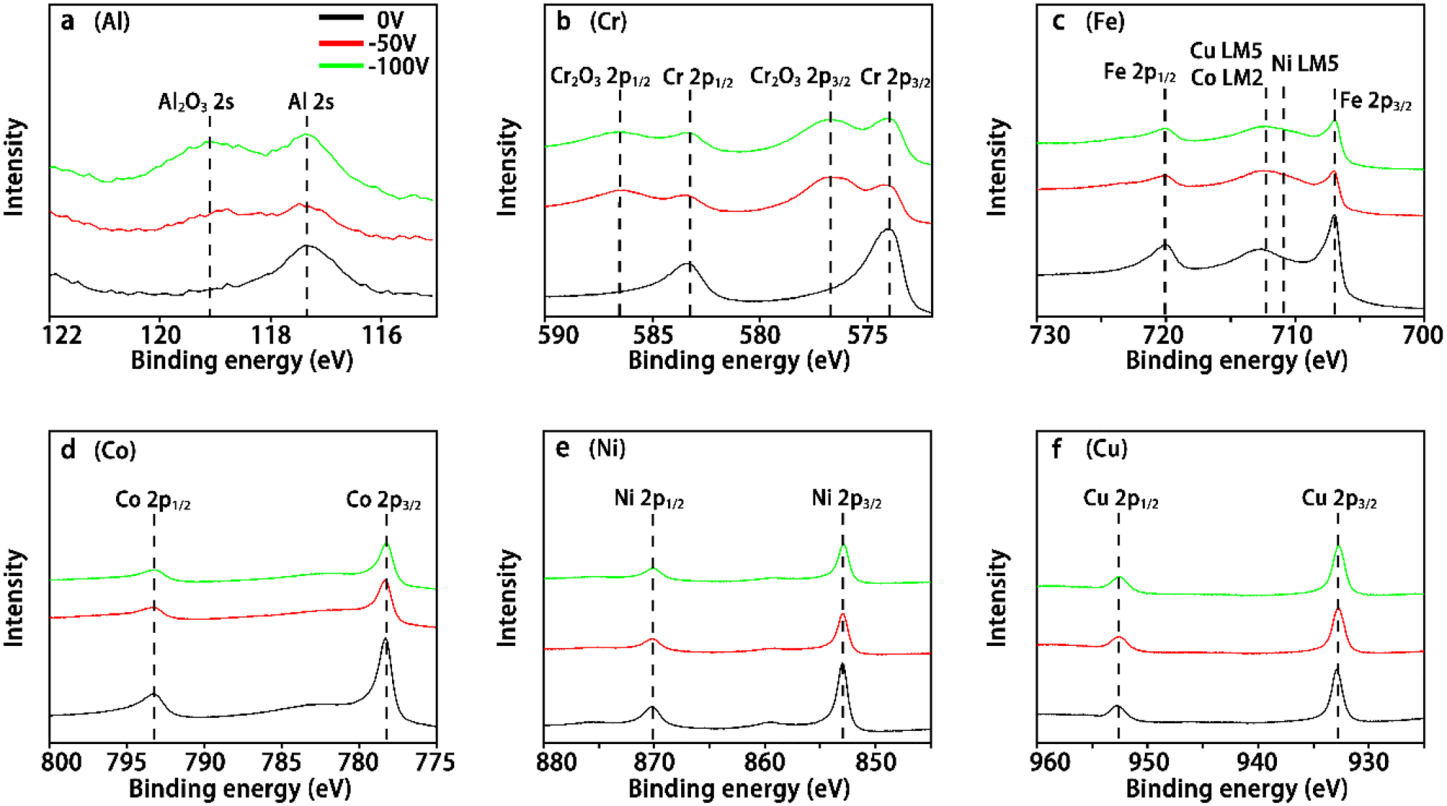
XPS high-resolution spectra of (**a**) Al-2 s; (**b**) Cr-2p; (**c**) Fe-2p; (**d**) Co-2p (**e**) Ni-2p; and (**f**) Cu-2p.

**Figure 8:**
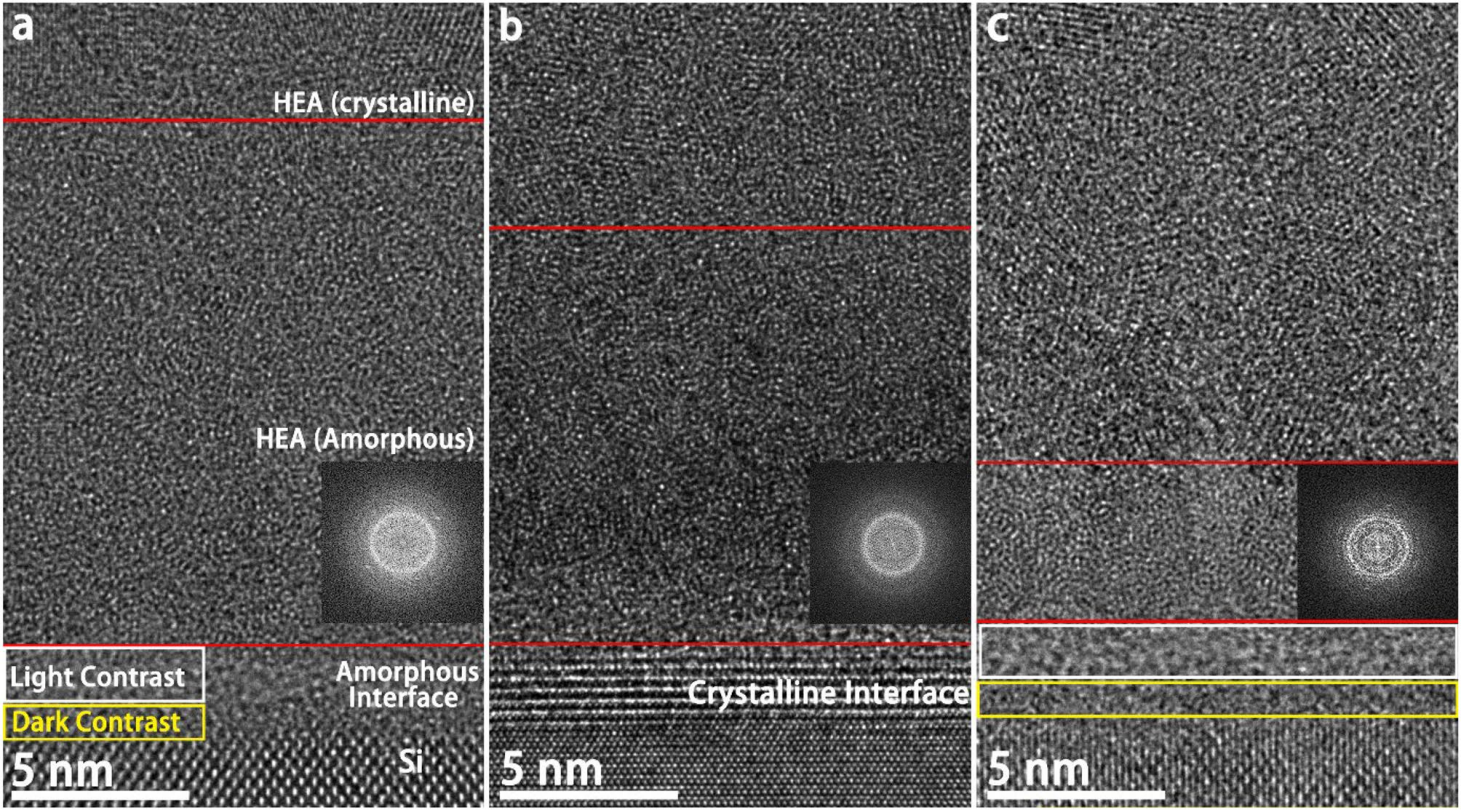
BF HRTEM of interface between Si and the HEA thin film. The insets are the Fast Fourier Transform (FFT) patterns from the region between the two red lines. (**a**) no substrate bias; (**b**) −50 V substrate bias; and (**c**) −100 V substrate bias.

It can be seen from Fig. 6 that the segregations of Al and Cu became more apparent as the substrate bias was increased from 0 V to −100 V (Fig. 7). The irregular-shaped enrichment regions of Al and Cu indicate that the segregations were resulted from both dendritic and cellular growth during the formation of columnar grains, as shown in Fig. 8^36,37^. The dendritic segregation was caused by the difference of elemental crystallization sequence (depending on the elemental melting point) at the stem and the branches. In this case, Al and Cu, with much lower melting points of 660.3 °C and 1084.6 °C respectively as compared to the other four metallic elements, crystallized later at the branches. Also, the segregation of Cu would be enhanced by the high positive mixing enthalpy of Cu with Cr, Fe, Co, and Ni (12, 13, 6, and 4 kJ mol^−1^, respectively) in binary systems, leading to intense repulsive interactions between Cu and these four elements^38,39^. The high cooling rate in this physical vapor deposition process led to inadequate adatom mobility, which further contributed to the non-uniform elemental distribution. Furthermore, constitutional supercooling simultaneously occurring at the vapor–solid interface promoted cellular growth^40^. As a result, cell boundaries would be enriched with elements that did not participate in grain growth, such as Al and Cu, and segregation became more distinct^40^ .

#### XPS surface chemistry

The surface chemistry of the AlCrFeCoNiCu_0.5_ thin films deposited with different biases was analyzed by XPS. The high-resolution spectra of each element’s photoelectron peak are shown in Fig. 7. The use of Al 2 s instead of the Al 2p orbital data was to avoid the overlap of the Cu 3p signals. To minimize the surface oxidation, the samples were stored in a desiccator and the XPS measurements were conducted within 24 h of the film deposition. Although the storage times for all the samples were kept the same, the spectra of Al and Cr reveal different levels of oxidation for different samples where signals of ${\mathrm{Al}}_{2}{\mathrm{O}}_{3}$ and ${\mathrm{Cr}}_{2}{\mathrm{O}}_{3}$ were found from the thin films deposited under a −50 V and −100 V substrate bias, respectively. The thin film deposited under a 0 V substrate bias only showed pure metallic Al and Cr signals, indicating a much lower level of oxidation state. The different oxidation rates of different samples are believed to be caused by differences in surface roughness, since lower roughness can generally lead to lower oxidation rate. Only metallic signals were observed in the spectra of Fe, Co, Ni, and Cu. A broad signal peak appeared between the Fe 2p_1/2_ and Fe 2p_3/2_ peaks, was likely due to the interference of Auger electrons from Cu LMM, Co LMM, and Ni LMM. The extent of oxidation for each element can be determined by the formation enthalpies of the different oxides. The formation enthalpies of ${\mathrm{Al}}_{2}{\mathrm{O}}_{3}$ and ${\mathrm{Cr}}_{2}{\mathrm{O}}_{3}$ are significantly lower than that of the other possible oxides. These naturally formed surface oxide layers are generally dense and stable, leading to good protective properties, such as oxidation and corrosion resistance, due to a significant diffusion barrier.

### Microstructure evolution of HEA thin films

The microstructure evolution of the HEA thin films deposited by the cathodic arc system under different substrate biases was investigated by HRTEM. From Fig. 8, it is evident that the microstructural texture of the HEA thin films can be divided into three regions (from the bottom interface to the bulk of the film), the interface between the silicon substrate and the thin film, the amorphous layer (delineated by the two red lines) and the polycrystalline region. The microstructure evolutions of these parts are discussed in the following sections.

#### Film-substrate interface

As shown in Fig. 8, the film-substrate interface displays a phase transition from amorphous (Fig. 8a) to crystalline (with lattice points being clearly observed in Fig. 8b) and then back to amorphous (Fig. 8c) as the substrate bias increased. The interface region of the sample deposited under a substrate bias of −100 V is slightly thicker than those deposited under 0 and – 50 V. Also, there is no clear thickness difference between the interfacial regions of the samples deposited under 0 and – 50 bias voltages. As discussed in Section 3.3.2, an Al_2_O_3_ compound interface is likely to exist between the silicon substrate and the thin film, but whether it is a crystalline or amorphous depended on the energy available for atomic diffusion. In Fig. 8a, the formation of an amorphous interface could be attributed to the rapid quenching effect during the physical vapor deposition, which provided insufficient thermal energy for atom diffusion to form a crystalline structure^17^. While many nucleation sites formed at the surface of the silicon substrate at the beginning of deposition due to the high supercooling degree of the interface between the metallic vapor and the solid silicon surface, these nucleation sites cannot grow into crystallites as the thermal energy (converted from ion kinetic energy) was rapidly lost by conduction through the silicon wafer because of rapid quenching^41^. Also, it is worth mentioning that a contrast difference at the interface (as marked in white and yellow rectangles in Fig. 8a,c) can be observed. The EDS mapping showed that while all elements appeared in the interface, they were separated as metallic-atom-rich and silicon-O-rich regions. The difference in elemental distribution was a result of atomic diffusions in opposite directions caused by the formation of compressive stress field due to the elastic combination between the HEA thin film and the silicon substrate. Additionally, the contrast variation at the interface as shown in the HRTEM images can be explained by the different electron diffraction capacity between silicon and other metallic atoms. This phenomenon is consistent with the results of EDS mapping, further supporting the existence of Kirkendall effect during thin film deposition.

A crystalline compound interface can be clearly observed in Fig. 8b. The increase in ion kinetic energy due to the application of the −50 V bias promoted adatom mobility at the interface that drove the crystallization^42^. In addition to the enrichment of Al and O at the compound interface, relatively weaker signals of other elements can be observed in Fig. 6. The crystalline compound interface is likely an Al_2_O_3_-based secondary solid solution. Based on the understanding of the cathodic arc system, it can be inferred that ion bombardment and the intensity of interfacial stress field would be simultaneously strengthened as the ion kinetic energy was enhanced. The compound interface still remained in crystalline state when the substrate bias of −50 V was applied during the deposition. This indicates that the strong ionic bond is not easy to break due to high bond energy, thereby contributing to the stability of the interface. However, the interface transformed to an amorphous state when the substrate bias was set to −100 V. This transformation from crystalline to amorphous state can be explained by two phenomena. On the one hand, the as-formed crystalline compound interface can be destructed by violent ion bombardment. On the other hand, the higher stress generated at the interface further strengthened the Kirkendall effect that boosted amorphization, leading to a relatively thicker diffusion layer. In conclusion, the change in substrate bias varied the Kirkendall effect, leading to the structural variations of Si-HEA interface.

#### Amorphous region

The regions in Fig. 8 between the two red lines were confirmed as amorphous by Fast Fourier Transform (FFT) images, and the thickness of the layer decreased as the substrate bias increased. Both the amorphous regions in Fig 8a and 8b are uniform, but the trace of columnar grains can be clearly observed in Fig. 8c. This phenomenon could be interpreted by the impact of nucleation rate and atomic diffusion rate on film deposition at different stages. More nucleation sites would be formed at the Si-HEA thin film interface at a lower substrate bias due to greater supercooling as there was less thermal energy being converted from the ion kinetic energy. However, a low atomic diffusion rate simultaneously inhibited the nuclei growth to grains^41^. The thin film temperature increased with the continuous deposition caused by heat accumulation over the entire sample because the amorphous layer acted as a barrier for thermal conduction through the silicon substrate due to isotropy^43,44^. The increase of thin film temperature with increased ion energy due to the increasing of bias provided more thermal energy to drive atom diffusion, which promoted growth and crystallization of the nucleation sites. Therefore, higher substrate bias led to a higher heating rate, which reduced the thickness of the amorphous region because the required temperature for crystallization could be reached more quickly at the substrate bias of −100 V than at 0 V or −50 V.

In summary, the homogeneous amorphous region became thinner and tended to form column-shaped structures as the substrate bias increased. The supercooling degree of the vapor-solid interface was rapidly reduced when the Si-HEA thin film interface formed due to the increasing of temperature, resulting in a lower density of nucleation sites at a higher substrate bias. The nuclei grew into columnar grains because the growth speed along the direction perpendicular to the substrate surface is the largest due to its greatest thermal conduction rate ^45^. However, the growth of the columnar structures failed to reach a crystalline state, which can be attributed to insufficient atomic diffusion to fully crystalize due to the rapid quenching during the physical vapor deposition. Additionally, although no obvious trace of columnar grains could be observed in Fig. 8b, the corresponding EDS mapping in Fig. 6b shows the appearance of Al and Cu segregation. This further explains the transit of the homogeneous amorphous region to the column-shaped region with the increasing of the substrate bias since dendritic growth and cellular growth are two typical mechanisms for columnar grain formation^46,47^.

#### Polycrystalline region

**Figure 9:**
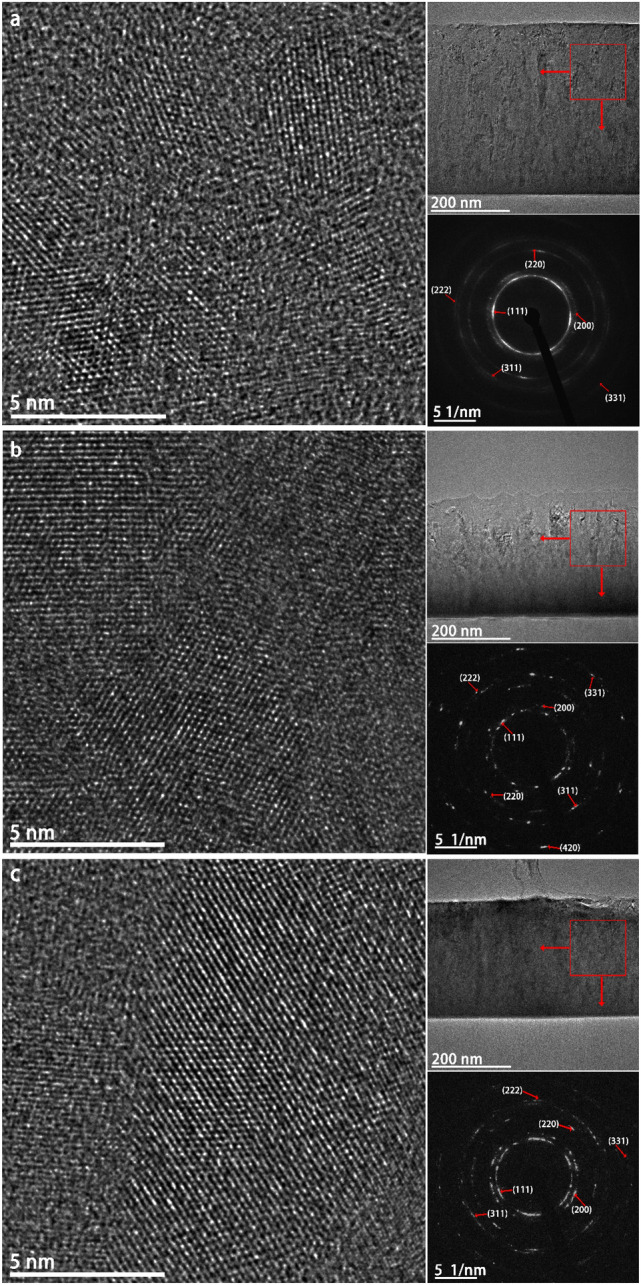
Cross-sectional TEM analysis of the HEA thin films fabricated with (**a**) no substrate bias; (**b**) −50 V substrate bias; and (**c**) −100 V substrate bias (HRTEMs and EDPs were captured from the red square regions shown in the BF TEM images with low magnification).

The detailed analysis of the polycrystalline regions in different samples is presented in Fig. 9. Each panel includes a bright-field (BF) HRTEM image, a low magnification BF image and an electron diffraction pattern (EDP). The HRTEM images (of the regions marked by the red squres) show that all the polycrystalline regions consist of many small regular-shaped equiaxed grains of different sizes. The crystallization and growth of the equiaxed grains increased with the increasing of the atomic diffusion rate in the system. The size of the equiaxed grains varied with the level of substrate bias during the thin film deposition (4–5 nm for 0 V, 9–10 nm for −50 V, and 14–15 nm for −100 V). The grain size correlated to the nucleation site density. Ion bombardment led to smaller grain size, while high temperature resulted in larger grain size^48^.

Here the concept of ion-bombardment-induced thermal spike is introduced to describe the grain growth behavior affected by ion bombardment. Within a spike region, sputtering, displacement and diffusion of atoms occur simultaneously. The number of thermal spikes per unit area and per unit time is constant as it depends on the depositing flux, which is a function of the cathodic arc parameters and does not vary with substrate bias. However, the energy embodied in each spike increases with the ion bombardment energy, which increases with the increasing of substrate bias. As sputtering increases with increased ion bombardment energy, the number of atoms sputtered from the growing film per ion impact thermal spike increases with the increasing of the substrate bias. This leads to the reduction in deposition rate as noted earlier. The increase in displaced and diffused atoms as a result of higher energy spikes created by higher substrate bias promotes nucleation and thus increases the nucleation site density leading to smaller grain sizes. Additionally, the thin film temperature increases with on-going deposition due to heat accumulation, producing an adequate supply of thermal energy to provide the mobility needed to drive crystallization^49^. Therefore, the formation of equiaxed grains and the corresponding small grain size for each sample were due to the increased number of nuclei and the relatively uniform thermal conduction. However, higher substrate bias results in higher energy delivered in each thermal spike that leads to higher rate of temperature increase which inhibits nucleation and increases diffusion, making the average grain size larger. This could be the reason for the trend of increased grain size with the increasing negative substrate bias. More energetic ion bombardment induced by higher substrate bias results in increased ion penetration depth and therefore larger thermal spike range, which reduces the density of disordered atoms at the thermal spike edges. As a result, higher temperature caused by higher substrate bias provides more thermal energy for disordered atoms to overcome the energy barrier and participate in crystallization during grain growth, leading to the decrease of grain boundary formation and increase in crystal size. In conclusion, the grain size strongly depends on the nucleation rate, while crystallinity correlates to the atomic diffusion rate.

The three low-mag BF images in Fig. 9 clearly show the cross-sectional morphology of the thin films deposited at different substrate bias voltages. The measured thin film thicknesses of 430 nm, 350 nm and 275 nm (corresponding to a substrate bias of 0 V, −50 V, and −100 V, respectively) based on the BF images are consistent with the thicknesses obtained using the profilometer (Fig. 4). The reason for the film becoming thinner as the substrate bias increases is the ion etching effect, which increases due to increased sputtering with the increasing of the substrate bias, as has been discussed in Sect. 3.2.

It can also be observed that there are many irregular fringes shown in the BF images, indicating that there is high interfacial energy between adjacent grains due to high grain boundary density. When combined with the Moire fringes, the irregular fringes became more obvious. Furthermore, the electron diffraction analysis of selected area EDPs, taken from equiaxed grain regions, revealed that the thin films have a single face centered cubic (FCC) phase, which can be explained by the fact that the FCC phase is preferred when the Al atomic fraction is below 15%^50^. The calculated lattice parameters based on EDPs for samples deposited at substrate biases of 0 V, −50 V, and −100 V are slightly different, corresponding to 3.73 Å, 3.77 Å, and 3.83 Å, respectively. In order to identify and estimate the residual stress theoretically, the interatomic spacing from the lattice parameters of our AlCrFeCoNi ${\mathrm{Cu}}_{0.5}$ samples is compared with the interatomic potentials of the AlCrFeCoNi^51^ and the CrFeCoNiCu^52^ systems. It is noted that the stress level of all our samples is generally close to the saddle point of the interaction potential curve, indicative of low residue stress. Further, a slight tensile stress increase appeared with the increasing of substrate bias due to formation of more vacancies at the grain boundaries.

#### Surface roughness

**Figure 10:**
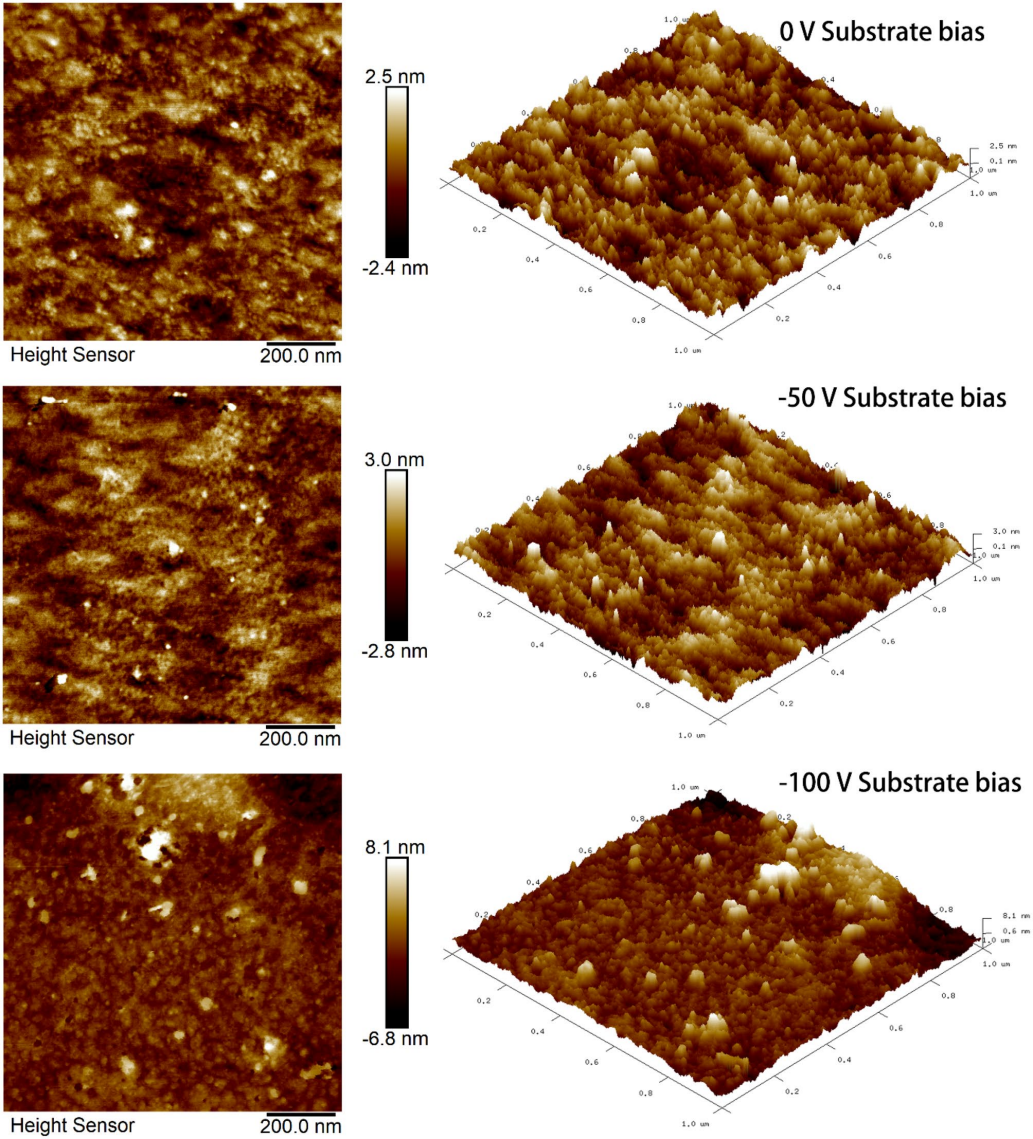
AFM analysis of the HEA thin films deposited at 0 V, −50 V, and −100 V substrate bias.

The surface roughness of the HEA thin film samples measured by AFM (Fig. 10) is consistent with our observations from the low-mag BF images shown in Fig. 9. The average surface roughness tended to increase from 0.72 ± 0.65 nm to 1.78 ± 1.38 nm corresponding to 0 V and −100 V of the substrate bias. The average roughness of the thin film grown at −50 V of substrate bias was 0.85 ± 1.1 nm. The surface roughness of a polycrystalline thin film produced by physical vapor deposition typically results from differences in the growth rate of grains with different orientations^35^. The low surface roughness obtained for the HEA thin films here is explained by the growth behavior of equiaxed grains, where the growth rates of various orientations are similar.

## Conclusions

AlCrFeCoNi ${\mathrm{Cu}}_{0.5}$ HEA thin films were fabricated by cathodic arc deposition under controlled ion energy by the application of different substrate biases of 0 V, −50 V, and −100 V. A deposition rate decline with increasing negative substrate bias was observed that was due to ion backscattering and sputtering, as predicted by TRIM simulations. The results from STEM-EDS revealed a reduced Al concentration in all samples compared to the cathode. This can be explained by preferential sputtering of elements with lower atomic mass with Al being the lowest in surface binding energy. A detailed study of the interface showed that a diffusion interface and a compound interface exist concurrently, and both are likely to contribute to improved adhesion. The segregation of Al and Cu tended to increase when a higher substrate bias was applied. This phenomenon is identified as a manifestation of both dendritic and cellular segregation occurring in columnar grain growth. The surface chemistry analysis indicated the natural formation of ${\mathrm{Al}}_{2}{\mathrm{O}}_{3}$ and ${\mathrm{Cr}}_{2}{\mathrm{O}}_{3}$ on the thin film surface. The presence of these stable oxide layers suggests that these films would possess both good chemical and thermal protection properties.

HRTEM observation suggested that the microstructure of each HEA thin film sample can be classified into three parts, the interfacial, amorphous, and polycrystalline regions. The interfacial region formed due to the mixing of atoms from the substrate with those deposited in the growing film. Compounds with favorable enthalpy such as alumina are found here, and the extent of the region depends on the energy of the deposited species and therefore the substrate bias. The formation of crystalline or amorphous state at the interface not only depends on the energy for atomic diffusion but also on the extent of ion bombardment. The presence of an amorphous region on top of the interface layer can be attributed to rapid quenching leading to a lack of thermal energy to drive nucleation and crystallization. The morphology transition and thickness variation of this amorphous region caused by the increasing substrate bias are indicative of the impact of increasing temperature on the nucleation and atomic diffusion rates. The formation of equiaxed grains in the next layer of the film is related to nucleation and atomic diffusion as well. The HRTEM of the polycrystalline region showed the tendency of equiaxed grain size to enlarge with increasing substrate bias due to the increase in crystallization facilitated by increasing growth temperature. The EDPs taken from crystal grains in this region showed only FCC phase crystal grains in all of the samples with only slight variations in lattice parameter as observed with the increasing of the substrate bias. These changes in lattice parameter were attributed to changes in internal stress associated with the increasing of ion energy and growth temperature as a result of increasing substrate bias.

## Supplementary Information

Supplementary Information.

## Data Availability

All data generated or analysed during this study are included in this published article and its supplementary information file.
