# Supplementary material for: The fabrication and growth mechanism of AlCrFeCoNiCu0.5 HEA thin films by substrate-biased cathodic arc deposition
Source: Sci Rep. 2023 Jan 5;13:198. doi: 10.1038/s41598-022-26232-9 (PMC9814579; doi:10.1038/s41598-022-26232-9)
Supplement: Supplementary file 1 — Supplementary Information. [file 41598_2022_26232_MOESM1_ESM.docx]

Supplementary Information

**The fabrication and growth mechanism of AlCrFeCoNiCu0.5 HEA thin films by substrate-biased cathodic arc deposition**

Hong Zhao^1,†^, Zhong Zheng^1,†^, Behnam Akhavan^2,3,4, 5^, Kostadinos Tsoutas^3,4,*^, Lixian Sun^6^, Haoruo Zhou^7^, Marcela M Bilek^2,3,4,*^, Zongwen Liu^1,2,*^

^1^School of Chemical and Biomolecular Engineering, The University of Sydney, NSW 2006, Australia

^2^The University of Sydney Nano Institute, The University of Sydney, Sydney, NSW 2006, Australia

^3^School of Physics, The University of Sydney, Sydney, NSW 2006, Australia

^4^School of Biomedical Engineering, The University of Sydney, Sydney, NSW 2006, Australia

^5^School of Engineering, University of Newcastle, Callaghan, 2308 NSW, Australia

^6^Guangxi Key Laboratory of Information Materials, Guangxi Collaborative Innovation Center of Structure and Property for New Energy and Materials, School of Material Science & Engineering, Guilin University of Electronic Technology, Guilin, 541004, PR China.

^7^School of Aerospace, Mechanical and Mechatronic Engineering, The University of Sydney, NSW 2006, Australia

*Corresponding authors

†These authors contributed equally to this work

**Table S1.** Input parameters of each element applied for TRIM modelling^1–3^.

| Element | Displacement energy (eV) | Lattice binding energy (eV) | Surface binding energy (eV) |
| --- | --- | --- | --- |
| Al | 25 | 3 | 4.85 |
| Cr | 40 | 3 | 5.33 |
| Fe | 40 | 3 | 5.45 |
| Co | 40 | 3 | 5.52 |
| Ni | 40 | 3 | 5.55 |
| Cu | 30 | 3 | 4.93 |


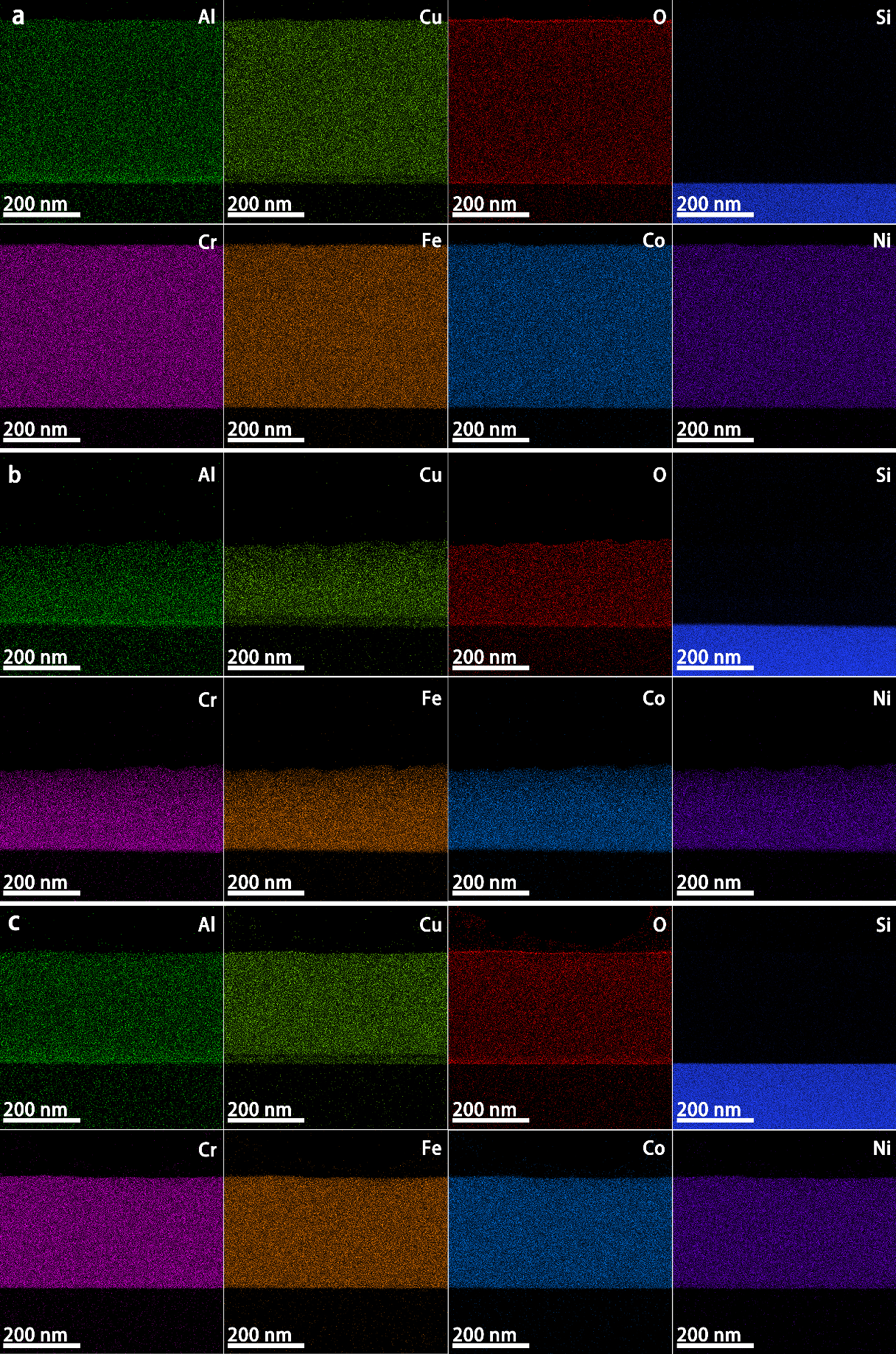


**Figure S1.** Cross-sectional view of STEM-EDS mapping of HEA thin films with various substrate bias. (a) 0V of substrate bias (b) -50V of substrate bias and (c) -100V of substrate bias.





**Figure S2.** Hardness and elastic modulus of HEA thin films with various substrate bias tested by nanoindentation (Hysitron TI 850 TriboIndentor). Each sample was tested 16 times with a indentation depth between 1/10 and 1/3 of the corresponding thin film thickness.
